# Fulminant Liver Failure after Treatment with a Checkpoint Inhibitor for Gastric Cancer: A Case Report and Review of the Literature

**DOI:** 10.3390/jcm12144641

**Published:** 2023-07-12

**Authors:** Miriam Dibos, Johanna Dumoulin, Carolin Mogler, Silke Wunderlich, Maximilian Reichert, Sebastian Rasch, Roland M. Schmid, Marc Ringelhan, Ursula Ehmer, Tobias Lahmer

**Affiliations:** 1 Department of Internal Medicine II, School of Medicine, University Hospital Rechts der Isar, Technical University of Munich, Ismaninger Str. 22, 81675 Munich, Germany; miriam.dibos@mri.tum.de (M.D.);; 2 Department of Pathology, School of Medicine, University Hospital Rechts der Isar, Technical University of Munich, Ismaninger Str. 22, 81675 Munich, Germany; 3 Department of Neurology, School of Medicine, University Hospital Rechts der Isar, Technical University of Munich, Ismaninger Str. 22, 81675 Munich, Germany

**Keywords:** nivolumab, checkpoint inhibitor, hepatic failure, encephalitis

## Abstract

Nivolumab is a promising monoclonal antibody inhibitor of programmed death-1, a protein on the surface of T-cells. As such, it is approved for use in patients with multiple advanced malignancies and can significantly elongate progression-free survival. However, monoclonal antibody inhibitors can lead to adverse hepatic reactions, which in rare cases result in further hepatic damage. Herein, we present a case of a patient with locally advanced gastric carcinoma treated with fluorouracil, oxaliplatin, docetaxel and the checkpoint inhibitor nivolumab. Five months after her first dosage of nivolumab and without a preexisting liver disease, she presented with transaminitis. During the course of her stay, the patient developed status epilepticus, which required mechanical ventilation followed by fulminant hepatic failure. A subsequent liver biopsy revealed severe liver damage with extensive confluent parenchymal necrosis corresponding to checkpoint-inhibitor-induced hepatitis. Alternative reasons for this hepatic failure were ruled out. Despite aggressive therapeutic interventions including corticosteroids and plasma exchange, the patient died due to liver failure. Although hepatic failure is rarely seen in patients with checkpoint inhibitor therapy, it requires early awareness and rapid intervention.

## 1. Introduction

Drug-induced liver failure is the most common cause of acute liver failure in Western Europe and the United States, while virus infections (mainly hepatitis A and E) are primarily responsible for acute liver failure in the developing world [1,2]. Although in many cases of drug-induced liver failure, the actual causative drug is not identified, the most common causative drug is reported to be acetaminophen (Paracetamol) [2,3]. Less common causes for liver failure include Budd–Chiari syndrome, Wilson’s disease or secondary hepatic failure due to acute ischemic hepatocellular injury in patients with cardiac or respiratory failure [1]. The latter is usually reversible when cardiorespiratory stabilization is reached [1]. For liver failure, early liver transplantation can be a crucial therapy option, as only 50–60% of patients with drug-induced liver failure survive without a liver transplant [4,5].

Nivolumab is a fully human IgG4 monoclonal antibody inhibitor of programmed death-1 (PD-1). It has been approved for and revolutionized the treatment of patients with multiple advanced malignancies [6,7]. These include melanoma, non-small cell lung cancer, renal cell cancer, urothelial carcinoma and gastric or gastro-esophageal junction cancer [6,7]. Nivolumab has shown substantial progression-free survival and a considerable overall survival benefit [6,7]. Monoclonal antibody inhibitors such as nivolumab inhibit immune checkpoint receptors and therefore enhance T-cell-enhanced antitumor activity [8,9,10]. One of the earliest manifestations of the toxicity of checkpoint inhibitors is skin rash; other later side effects include diarrhea and colitis (in about 20% of the patients) [11]. Nivolumab can also lead to hepatic damage, varying from transaminitis to liver injury due to an idiosyncratic reaction based on an autoimmune response [12]. Typical side effects usually occur within 8 to 12 weeks [11].

We report a case of suspected drug-induced liver failure in a patient with a gastric carcinoma of the signet ring cell type with peritoneal metastases, who developed fulminant hepatic failure due to treatment with the checkpoint inhibitor nivolumab.

## 2. Case Presentation

**Figure 1:**
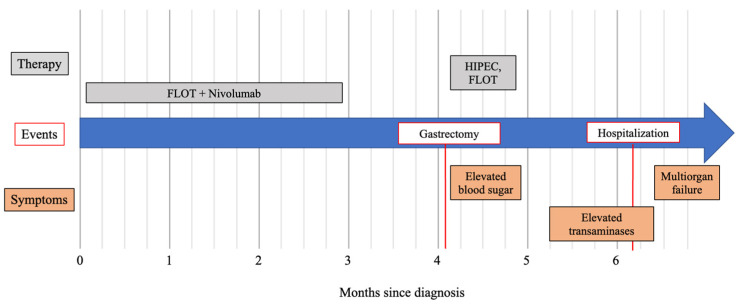
Timeline of the patient’s diagnosis, treatments and symptoms. Symptoms (in orange), therapy (in gray) and events (in red) are described in a timeline measured in months since diagnosis. Events are illustrated by red bars; symptoms or therapy of longer persistence are indicated by bars. FLOT: fluorouracil, oxaliplatin and docetaxel; HIPEC: hyperthermic intraperitoneal chemotherapy.

A female aged 51 presented to our outpatient clinic with an unclear hepatopathy. Six months prior, she had been diagnosed with a locally advanced gastric carcinoma. Except for the gastric carcinoma, the patient did not have any other pre-existing medical conditions. Previous treatment of the gastric carcinoma had included neoadjuvant treatment with six cycles of fluorouracil, oxaliplatin and docetaxel (FLOT). Five out of these six cycles of FLOT had included the checkpoint inhibitor nivolumab. This neoadjuvant treatment was followed by a gastrectomy with roux-en-y anastomosis and hyperthermic intraperitoneal chemotherapy (HIPEC). The postoperative treatment comprised two more cycles of FLOT without nivolumab. This was due to the fact that the patient developed elevated blood sugar, which was suspicious of antibody-induced diabetes mellitus. Though the patient did not have a history of chronic liver disease, she developed elevated liver values three months after the last nivolumab dose. Due to those elevated liver values, she presented to our outpatient clinic. A timeline of symptoms, events and therapy is illustrated in Figure 1.

**Figure 2:**
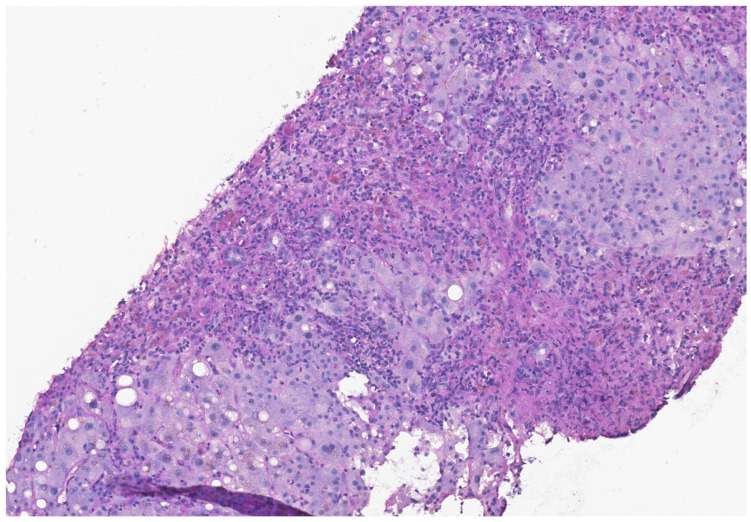
Representative image (diastase PAS) showing confluent and bridging necrosis with inflammatory responses (macrophages, granulocytes).

On admission, her total bilirubin level was 4.2 mg/dL, alkaline phosphatase was 282 U/L, alanine transaminase (ALT) level was 1177 U/L, international normalized ratio (INR) was 2.1, prothrombin time was 45 s, ammonia level was within its normal limit, and albumin level was 3.0 mg/dL. Hepatitis A, B and C, cytomegalovirus, Epstein–Barr virus, adenovirus and human immunodeficiency virus, as well as urine and blood cultures, were all negative. Ferritin levels were within their normal limits. As the elevated bilirubin level suggested cholestasis, we performed abdominal sonography and could exclude biliary obstruction. A transjugular liver biopsy revealed severe liver damage with extensive confluent parenchymal necrosis with chronic inflammation. This parenchymal necrosis is compatible with checkpoint-inhibitor-transmitted hepatitis (see Figure 2).

With the suspicion of hepatitis related to checkpoint inhibitor treatment, the patient was started on high-dose prednisone therapy according to the literature (60 mg prednisone per day) [13,14].

Ten days after being admitted to our general ward, she developed a non-convulsive status epilepticus and was transferred to our intensive care unit (ICU). The neurological symptoms were observed by an experienced neurologist and were completely reversible upon treatment (4 mg midazolam and 4.5 g levetiracetam). An electroencephalogram (EEG) showed no evidence of seizure activity. Nonetheless, it showed diffuse slowing, which is suggestive of severe nonspecific encephalopathy. Magnetic resonance imaging (MRI) and the results of lumbar puncture (cell count, microbiologic and virologic testing) were unremarkable. Over the course of her stay, she developed another non-convulsive status epilepticus, which required protective intubation due to her deteriorating mental status. Subsequently, EEG and cerebral computer tomography (cCT) were repeated. The EEG showed no evidence of seizure activity, and the cCT did not show any signs of cerebral ischemia or hemorrhage. The patient then developed fulminate liver failure and, consequently, multi-organ failure (total bilirubin level was 6.5 mg/dL, alkaline phosphatase was 264 U/L, ALT level was 428 U/L, INR was 4.1, Quick was 17%, prothrombin time was >180 s, albumin was 2.5 g/dL, factor V was 18%). Her circulation required high catecholamine doses (noradrenalin up to 12 ug/kg/min, vasopressin up to 2 units/h). Furthermore, she developed an acidosis with a pronounced lactatemia of up to 22 mmol/L. Due to their duration of several days, the acidosis and lactatemia could not be explained by the seizure alone. Therefore, they were interpreted as part of the liver failure. Because of the acidosis, she was then treated with continuous veno-venous hemofiltration, which temporarily decreased the lactatemia down to 2.2 mmol/L. Furthermore, she received intermittent plasmapheresis because of the massive coagulation disorder. Thoracic and abdominal computer tomography (CT) showed an increasing quantity of ascites and sustained hepatic perfusion. Furthermore, CT excluded mesenteric ischemia, ileus and other acute pathologies. Despite maximal ICU treatment, the patient died three days after intubation. A request for autopsy was refused by the patient’s family.

## 3. Discussion

Immune-related adverse events related to checkpoint inhibitors include a broad spectrum of symptoms and are a common side effect of PD-1 antibody inhibitors. This is due to the potential exaggerated and uncontrolled immune response upon influencing endogenous immunologic tolerance [9]. A meta-analysis analyzing 20,128 patients revealed at least one adverse event in 66% of the patients [10]. Especially endocrine functions, skin and digestive tract are most frequently affected [15]. The patient described in our case report first developed endocrine, then hepatic and neurological symptoms. Endocrine adverse effects included elevated blood sugar values. Hepatic side effects were first shown by transaminitis, later hepatic failure and potentially hepatic encephalopathy. Neurological symptoms included non-convulsive status epilepticus and reduced vigilance. It remains indeterminate whether the neurological symptoms should be interpreted as a distinct autoimmune process or whether it was a secondary consequence of the hepatic failure (i.e., as a manifestation of hepatic encephalopathy). Hepatic encephalopathy is a reversible symptom due to reduced liver function. It is characterized by a spectrum of neurological and psychiatric abnormalities. The symptoms include a characteristic flapping tremor (asterixis), confusion, disorientation and reduced consciousness. Laboratory findings can include elevated ammonia levels. Treatment options include laxative measures (i.e., lactulose which helps convert ammonia to non-absorbable ammonium in the gastrointestinal system) [16]. Hepatic encephalopathy can lead to hepatic coma and ultimately to death. Our patient did not show flapping tremor, disorientation and confusion. Her laboratory chemistry showed normal ammonia. Furthermore, the symptoms did not disappear after laxative measurements. Neurological symptoms after checkpoint inhibitor treatment, however, are less common adverse events and typically include nonspecific symptoms such as headache and dizziness in up to 6.1% of the patients [15]. A case series by Larkin et al. described an incidence of 6 out of 376 patients with the rare complication of encephalitis after checkpoint inhibitor treatment [17]. In this paper, the median time to onset was 45 days [17]. As symptoms vary from altered mental status, headache, fever, (generalized or focal) and weakness to seizures, the clinical diagnosis may be difficult to make [15]. MRI can demonstrate non-specific inflammatory changes such as temporal lobe enhancement, but it can also be normal, while EEG may show seizures and nonspecific patterns or be unremarkable [18]. As recommended, we performed lumbar puncture, MRI and EEG. In our case, the results of MRI and the lumbar puncture were unremarkable. She developed a sudden deterioration of consciousness as part of a non-convulsive status epilepticus, which was first reversible upon treatment. Therefore, we evaluated the neurological symptoms either as part of a liver-failure-induced hepatic encephalopathy or as part of a checkpoint-inhibitor-induced encephalopathy. As the symptoms did not improve after laxative measurements, we rather interpreted the symptoms as part of a checkpoint-inhibitor-induced encephalopathy.

After introducing the spectrum of general adverse events with a focus on neurological symptoms, hepatic adverse events are discussed. To further address the hepatic adverse events of immune checkpoint inhibitors (i.e., ipilimumab, nivolumab, pembrolizumab, tremelimumab and pidilizumab), several meta-analyses analyzed the risk of elevated transaminases after the treatment with aforementioned antibodies [19,20]. These meta-analyses showed an increased risk of transaminitis and, less commonly, hyperbilirubinemia after the treatment with checkpoint inhibitors [19,20]. Looking at the timeline of adverse events, early onsets typically include skin rash or pruritus, while adverse events with a later onset can include colitis and liver toxicity [11]. Transaminitis typically occurs up to 8 to 12 weeks after the initiation of the treatment [20,21,22,23]. Nonetheless, elevated transaminases and hepatitis have also been reported up to 21 months after the initiation of the treatment [12,22]. Xu et al. searched the US Food and Drug Administration Adverse Event Reporting System (FAERS) database for events between 2015 and 2021 with an association between immune checkpoint inhibitors and hepatic failure [24]. They classified different checkpoint inhibitors into three different categories: PD-1 inhibitors, programmed cell death-1 ligand 1 (PD-L1) inhibitors and cytotoxic T lymphocyte-associated protein 4 (CTLA-4) inhibitors [24]. Of the 9,647,655 cases in the FAERS database, they found hepatic failure in 0.19% of the patients (18,454/9,647,655) with an association of 654 cases with checkpoint inhibitor treatment [24]. Nivolumab was associated with the highest rate of hepatic adverse events, followed by ipilimumab and pembrolizumab. The median time from first treatment to hepatic failure was 38 days [24]. A total of 72.3% of the adverse events occurred within the first 3 months [24]. Most of these cases had poor outcomes, with a mortality rate of 68.7% [24]. In our case, hepatopathy occurred five months after the first dose of nivolumab (see Figure 1). A liver biopsy can be a helpful tool to help rule out alternative reasons for liver injury. Nonetheless, it is not a mandatory diagnostic tool, and, thus, it should not delay therapy [25]. Hepatic histologic findings of checkpoint-inhibitor-induced hepatitis are sparse in the literature and vary depending on the type of checkpoint inhibitor (PD-1, PD-Ligand 1 or anti-cytotoxic T-lymphocyte antigen 4 (CTLA-4)). CTL-4 inhibitors tend to cause granulomatous hepatitis with central vein endotheliitis and fibrin ring granulomas [26]. PD-1 (such as nivolumab) and PD-Ligand 1 inhibitors show less various histopathological findings, predominantly lobular hepatitis [26,27]. In our case, a severe hepatitis with bridging necrosis was observable. The strong association with hepatic failure upon checkpoint inhibitor treatment (especially with nivolumab) and the poor outcome further emphasize the need for close and regular monitoring of these patients.

For the above-presented hepatic adverse events, only the following therapy options currently exist. Immune-checkpoint-inhibitor-related toxicities are commonly rated on five grades of ascending symptoms (asymptomatic: grade 1, moderate: grade 2, severe: grade 3, life-threatening: grade 4 and death: grade 5) [14]. After excluding other potential reasons for impaired liver function (such as hepatitis, EBV, CMV, HIV, alcohol-induced hepatitis and others), patients with a grade 2 rating or higher liver function abnormalities should receive immediate treatment with steroids for 4–6 weeks [14,25]. Patients with a grade 3 rating or higher liver function test should also be evaluated for inpatient hospital admission, a liver biopsy should be performed, and checkpoint inhibitor treatment should be discontinued [14,25]. Patients without response to treatment after 3 days should be considered for second-line agents such as immunosuppressive drugs like mycophenolate mofetil, tocilizumab, tacrolimus, azathioprine or cyclosporine [14,25]. Another therapy option addresses the enhanced T-cell activity after checkpoint inhibitor treatment. Some studies suspected the T-cell response to the antibody treatment with a persisting production of autoantibodies to be the underlying cause of adverse events [9,28]. In those cases, liver biopsies are abundantly filled with T lymphocytes, and specific “T-cell-depleting” antibodies could be a therapy option. Polyclonal antibodies such as antithymocyte globulins (ATG) deplete T lymphocytes but also other key cells such as B lymphocytes, regulatory T lymphocytes and natural killer T lymphocytes [29]. They, therefore, transmit a general immunosuppressive activity and are usually used in the treatment of acute rejection in solid-organ rejection, graft-versus-host disease and various autoimmune diseases [29]. As we did not perform T-cell-marker-specific staining (as CD4 and CD8), and it is not a routine therapy option in checkpoint-inhibitor-transmitted hepatic failure, we did not consider this therapy option. Nonetheless, ATG therapy may be another future therapy option.

Nonetheless, liver transplantation remains the only definite treatment option for fulminant liver failure, but donor livers are sparse, and treatment is expensive. Furthermore, liver transplantation is not an eligible therapy option for cancer patients.

## 4. Conclusions

Checkpoint inhibitor therapy fundamentally expanded treatment options for multiple malignancies in both metastatic and adjuvant settings. The incidence of immune-related adverse events remains low but will increase with the broader use of checkpoint inhibitors. Therefore, this case report highlights the necessity for an increased awareness of a broad spectrum of adverse events even several months after checkpoint inhibitor treatment and presents various therapy options for this condition.

## Data Availability

No new data were created or analyzed in this study. Data sharing is not applicable to this article.
